# Tumor Immune Microenvironment and Its Related miRNAs in Tumor Progression

**DOI:** 10.3389/fimmu.2021.624725

**Published:** 2021-05-18

**Authors:** Yingying Xing, Guojing Ruan, Haiwei Ni, Hai Qin, Simiao Chen, Xinyue Gu, Jiamin Shang, Yantong Zhou, Xi Tao, Lufeng Zheng

**Affiliations:** School of Life Science and Technology, China Pharmaceutical University, Nanjing, China

**Keywords:** miRNA, tumor progression, tumor immune microenvironment, tumor-associated macrophages, dendritic cells, natural killer cells, myeloid-derived suppressor cells

## Abstract

MiRNA is a type of small non-coding RNA, by regulating downstream gene expression that affects the progression of multiple diseases, especially cancer. MiRNA can participate in the biological processes of tumor, including proliferation, invasion and escape, and exhibit tumor enhancement or inhibition. The tumor immune microenvironment contains numerous immune cells. These cells include lymphocytes with tumor suppressor effects such as CD8+ T cells and natural killer cells, as well as some tumor-promoting cells with immunosuppressive functions, such as regulatory T cells and myeloid-derived suppressor cells. MiRNA can affect the tumor immune microenvironment by regulating the function of immune cells, which in turn modulates the progression of tumor cells. Investigating the role of miRNA in regulating the tumor immune microenvironment will help elucidate the specific mechanisms of interaction between immune cells and tumor cells, and may facilitate the use of miRNA as a predictor of immune disorders in tumor progression. This review summarizes the multifarious roles of miRNA in tumor progression through regulation of the tumor immune microenvironment, and provides guidance for the development of miRNA drugs to treat tumors and for the use of miRNA as an auxiliary means in tumor immunotherapy.

## Introduction

The tumor microenvironment (TME) facilitates growth of tumor cells *via* the interplay between tumor cells and surrounding cells. TME contains a variety of cells, including fibroblasts, endothelial cells, mast cells, and immune cells (1). Fibroblasts are predominantly related to vascular development and tumor cell proliferation (2), while endothelial cells are deemed to be an important source of cytokines that can boost TME homeostasis (3). Immune cells in the TME include T lymphocytes (CD4+ T lymphocytes and CD8+ T lymphocytes), natural killer (NK) cells, tumor-associated macrophages (TAM), dendritic cells (DC), myeloid-derived suppressor cells (MDSC), regulatory T cells (Treg), and neutrophils (4). Among them, T lymphocytes and NK cells inhibit tumor progression by recognizing tumor antigens or producing inhibitory products such as granzyme and perforin. TAMs are the most abundant type of immune cells and are categorized as M1 and M2 phenotypes. In the TME, TAMs are mainly of the M2 phenotype, which promote tumor proliferation and metastasis (5). DC function to present antigens, and those in the TME usually exist in an immature state and promote tumor progression (6). In addition, MDSC and Treg can promote tumor progression by exerting immunosuppressive effects (7). Many studies have shown that granulocytes, especially neutrophils, are also enriched in the TME. Tumor-related neutrophils usually have the N2 phenotype and promote tumor progression (8). It is thus evident that all these complex immune cells are intricately linked to tumor cells and can affect tumor progression through their different functions.

MicroRNA (miRNA), as a non-coding small RNA, is produced from precursor RNA and is usually composed of 19-25 nucleotides that can target and regulate downstream gene expression (9). Studies have shown that miRNAs are associated with the regulation of sundry diseases, such as inflammation and cancer (10). Abnormal expression of miRNA can affect the development of tumors. For example, Let-7, miR-7, miR-9, and miR-29b miRNAs can promote breast cancer invasion and stemness (11). However, in hepatocellular carcinoma (HCC), miR-26a, miR-548l, and miR-34a suppress tumor growth by targeting the ST3GAL5 gene (12). In addition, miRNAs can regulate components of the TME, mainly various types of immune cells (13). This review systematically summarizes the effects of miRNA regulation on various immune cells that are engaged in tumor progression. The review will act as a tool basis for studying the interaction between immune cells and tumor cells and will highlight significant references that may facilitate the development of miRNAs as potential clinical treatments or auxiliary tumor immunotherapies.

## MiRNA Roles in the TME

### MiRNA Roles in Tumors

MiRNAs are involved in most aspects of cancer biology, such as apoptosis, proliferation, angiogenesis, invasion/metastasis, and tumor stemness. Some miRNAs display tumor suppressor effects, such as miR-29b, which inhibited the expression of antiapoptotic genes Mcl-1 and Bcl-2, and promoted spontaneous apoptosis of tumor cells (14). Other miRNAs affect the metastasis and invasion process of tumor cells. For example, miR-21 promoted the invasion and metastasis of colon cancer tumor cells by down-regulating Pdcd4 in colon cancer (15). In terms of affecting angiogenesis of tumor cells, miR-497 was down-regulated in human ovarian cancer tissue, and this downregulation was positively correlated with an increase in tumor angiogenesis. Further investigation of the mechanism found that miR-497 exerted its antiangiogenesis function mainly by inhibiting the expression of VEGFA, thereby impairing the PI3K/AKT and MAPK/ERK pathways mediated by VEGFR2 (16). Tumor cell stemness is crucial for maintaining the development and continuous differentiation of tumors, which can promote tumor cell metastasis. Overexpression of miR-107 in brain tumor stem cells can inhibit their proliferation and also down-regulate Notch2 protein, as well as stem cell markers such as CD133 and Nestin (17). Proliferation, epithelial-mesenchymal transition, and other processes related to tumors can also be regulated by miRNAs. The miRNAs directly or indirectly influence the behavior of tumor cells, thereby exhibiting roles in promoting or suppressing tumors.

In the TME, there is a close relationship between tumor cells and their surrounding immune cells, and miRNA is thought to connect these cell types. Abnormal expression of miRNA in tumor cells can affect the immune microenvironment. For example, miRNA can control tumor cells to produce chemokines or cytokines, which then affect the aggregation and expansion of immune cells. Contrarily, tumor cell miRNA can be transported *via* exosomes to immune cells, thus exosomes as an important tool for miRNA transport can also affect the immune microenvironment. The diverse roles of miRNA on different tumor cells will be discussed in the following sections.

### MiRNA Roles in TAM

TAM are known to participate in tumor cell growth, survival, invasion, metastasis, angiogenesis, inflammation, and immunoregulation. Moreover, studies have found that miRNA are also involved in the process of regulating TAM to affect tumors.

**Figure 1:**
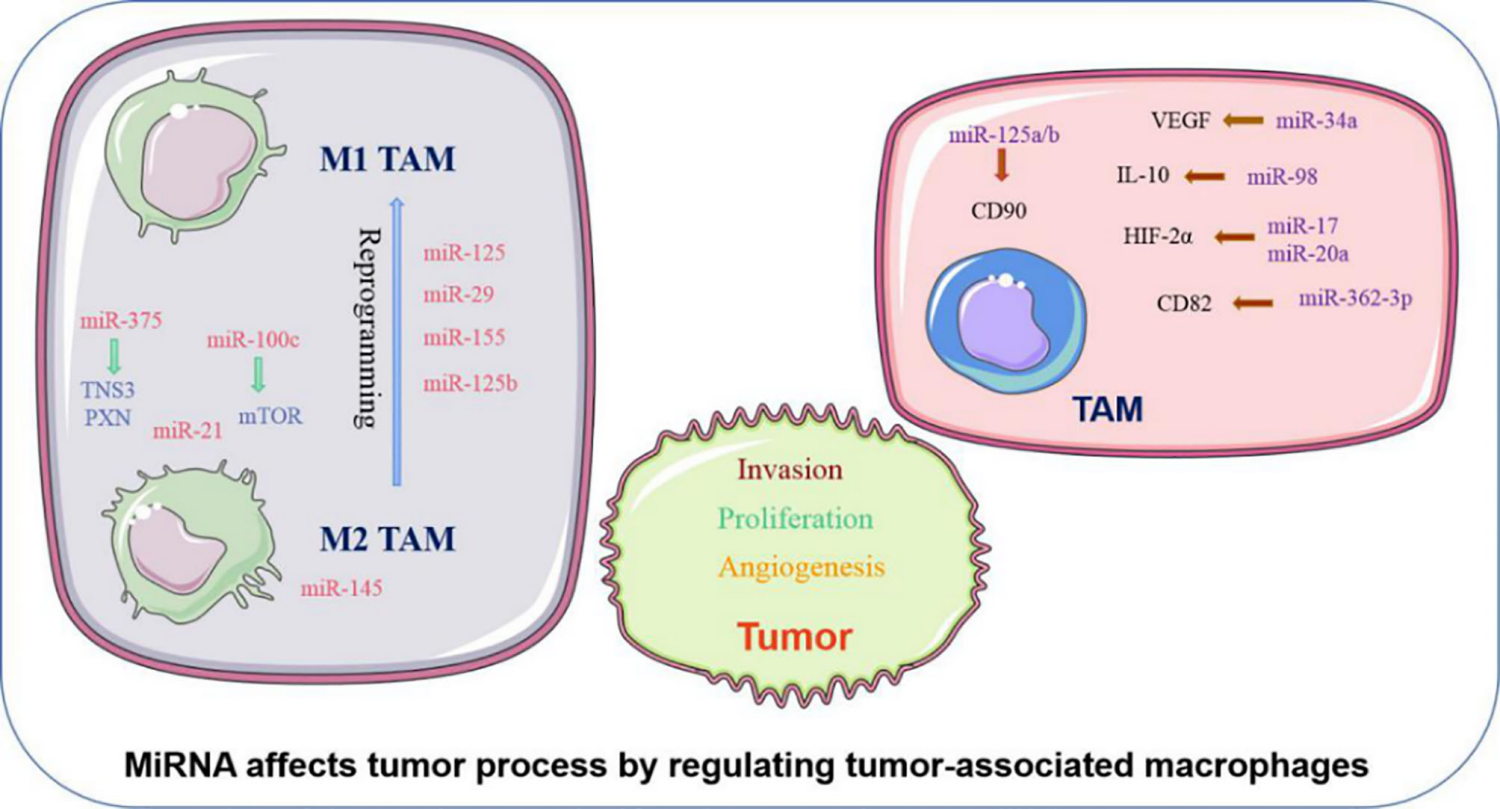
Abnormally expressed MiRNA in TAM can directly regulate its function. M1 and M2 TAM can be controlled by miRNA, which usually promote TAM differentiate into a certain phenotype, making them present M1 or M2 respectively. Certain M2 TAMs also undergo reprogramming after being affected by miRNA, causing to lost their tumor-promoting effect.

MiRNA can influence phenotypic changes and the polarization of macrophages (**Figure 1**). MiR-100c was observed to be overexpressed in TAM and could maintain the phenotype of TAM by targeting the mTOR signaling pathway. Moreover, intratumoral injection of the miR-100c antagomir abolished the metastasis and invasion capacities of breast cancer cells. This illustrated that miR-100c could promote tumor metastasis by maintaining the phenotype of TAM (18). MiR-375 is a bifunctional miRNA that is downregulated in HCC and gastric cancer but upregulated in breast cancer. Studies found that miR-375 released from tumor cells could be absorbed by macrophages through CD36 on the macrophage cell surface, and once inside, miR-375 altered macrophage infiltration and migration by targeting TNS3 and PXN. Thus, tumor-derived miRNA also influences the TAM phenotype (19). The polarization state of macrophages is intimately connected to tumor development. The previously studies have advanced that M1 macrophages primary involved in inflammatory response and antitumor immunity, while M2 macrophages customarily revealed the anti-inflammatory and pro-tumorigenic effects. MiRNA affects the polarization of TAM (**Figure 1**). In colorectal cancer, the cancer cell–derived extracellular vesicles containing miR-145 can polarize macrophages into the M2 phenotype and then promote tumor progression (20). In an analogous study on epithelial-mesenchymal transition (EMT)-mediated TAM activation, the EMT transcriptional factor Snail activated miR-21 in tumors to produce exosomes containing miR-21. These exosomes were absorbed by CD14+ human monocytes and suppressed the expression of M1 markers and increased the expression M2 markers, eventually accelerating tumor deterioration. This indicated that EMT influenced the polarization of M2 macrophages, which is mainly controlled by tumor-derived miR-21 (21). In addition to influencing the polarization of TAM, miRNA can reprogram macrophages in some cases, further demonstrating the role of miRNA as a protumor or antitumor effector. Recent studies in colon cancer cells revealed that human mutant p53(mutp53) cancer cells can reprogram macrophages under tumor supportive and anti-inflammatory conditions. Exosomes enriched in miR-1246 can be released by mutant p53(mutp53) colon cancer cells and are assimilated by macrophages (22). These macrophages then display cancer-promoting functions in an miR-1246-dependent manner. After reprograming TAM, miRNA can also recover the antitumor activity of macrophages. Certain miRNA such as miR-125, miR-29, and miR-155 are well known for promoting the repolarization of macrophages from the M2 phenotype to the M1 phenotype (23–25). In macrophages, miR-125b can be unregulated, and usually unleashes activating M1 macrophages, thereby promoting the antitumor effect. Research confirmed that when macrophage-specific HA-PEI nanoparticles were administered intraperitoneally to non-small cell lung cancer (NSCLC) mice, after transfection with miR-125b (26), the ratio of M1 macrophages increased markedly. Therefore, miRNAs have diverse and complex roles in the regulation of TAM characteristics and can govern the phenotype and polarization state of TAM to control tumor behavior.

In addition to affecting the above characteristics of TAM, miRNA can also influence the biological behavior of tumor cells by regulating the function of TAM (**Figure 1**). TAM can promote the proliferation and invasion of tumor cells. For example, TGF-β1 produced by TAM could downregulate the expression of miR-34a by upregulating VEGF, which ultimately enhanced the proliferation and invasion of colon cancer cells (27). Furthermore, miR-98 was observed to target and downregulate the expression of IL-10, thereby inhibiting TAM in promoting the metastasis and invasion of liver cancer cells (28). Tumor angiogenesis is critical to the continuous expansion of tumor cells. TAM can regulate the angiogenesis process of tumor cells because TAM can produce VEGF and other products of angiogenesis. It has been reported that HIF-2a promotes the expression of proangiogenic genes such as VEGFA and PDGFB when HIF-2a increases in TAM in non-hypoxic environments. MiR-17 and miR-20a are associated with regulating the promotion of HIF-2α on tumor cell angiogenesis (29). EMT is another critical behavior of tumor cells to promote their development, and can accelerate tumor cell metastasis and invasion. TAM are intricately linked with the EMT of tumor cells. TGF-β produced by M2 macrophages was reported to upregulate miR-362-3p by mediating the binding of Smad2/3 to the promoter region of miR-362-3p (30). Overexpressed miR-362-3p then regulated the expression of CD82, a molecule related to EMT, and subsequently promoted EMT of liver cancer cells (30). Recent reports suggest that exosomes produced by TAM promote the stemness of tumor cells. Analysis of the distribution of miRNAs in TAM exosomes found that miR-125a/b was significantly reduced. In addition, treatment of hepatic carcinoma cells with TAM exosomes or *via* transfection with miR-125a/b reduced the stemness of liver cancer cells by targeting CD90 and inhibited the proliferation of these cancer cells (31). In summary, miRNAs are instrumental in the regulation of tumor progression by TAM, and various miRNAs that influence the tumor process by regulating TAM are presented in (**Table 1**).

**Table 1:** MiRNA related with MDSC in tumor progression.

MiRNA	Status	Target	MiRNA’s regulating in TAM	Impact on tumor progression
MiR-155	Increased	FGF2	Enhance suppressive function	Suppress the cell viability, migration and invasion (32)
MiR-98	Increased	IL-10	Inhibit the function of TAM	Suppress migration and invasion of hepatocellular carcinoma cells (28)
Let-7a	Increased	STAT3 NF-κB	Suppress macrophage infiltrations and malignant phenotype	Decrease tumor growth (33)
MiR-30a	Increased	Twist1 Vimentin	Inhibit the function of TAM	Promote metastatic potential of bladder cancer (34)
MiR-25- 3p MiR-130b-3p MiR-425-5p	Increased	PTEN	Induce M2 polarization	Promote cancer metastasis (35)
MiR-125a	Increased	HIF-1α IRF4	Increase phagocytic activation	Repress tumor growth (36)
MiR-145	Increased	histone deacetylase 11	Polarize macrophage-like cells into the M2-like phenotype	Enlarge the tumor volumes (20)
MiR-142-3p	Increased	RAC1	Propofol stimulates TAM to secrete miR-142-3p	Inhibit HCC cell invasion (37)
MiR-21	Increased	PI3K/ AKT	Enhance the function of M2 TAM	Suppress cell apoptosis and confer cisplatin resistance in gastric cancer (38)
MiR-100	Increased	mTOR	Maintain the phenotype of TAMs	Promote tumor metastasis (18)
MiR-125b	Increased	CSF1/CX3CL1	Decrease the abundance of TAM	Alleviate the tumor growth (39)
MiR-1246	Increased		Reprogram macrophages to a tumor supportive and anti-inflammatory state	Promote colon cancer progression and metastasis (22)
MiR-375	Increased	TNS3 PXN	Enhance macrophage migration and infiltration	Develop a tumor-promoting microenvironment (19)
MiR-720	Increased	GATA3	Suppress M2 macrophage polarization	Inhibit migration of breast carcinomas (40)
MiR-221-3p	Increased	CDKN1B	Enhance function of TAM	Contribute to the proliferation and G1/S transition of epithelial ovarian cancers (41)
MiR-125b-5p	Increased	LIPA	Induce a tumor-promoting TAM phenotype	Promote tumor development (42)
MiR‐30c	Increased	REDD1	Promote M1 macrophage differentiation	(43)
MiR‐362‐3p	Increased	CD82	M2 macrophages mediate overexpression of miR‐362‐3p	Promote epithelial‐mesenchymal transition in hepatocellular carcinoma cells (30)
MiR-155	Decreased	C/EBPb	Promote tumor-activated monocytes to produce cytokine	(44)
MiR-34a	Decreased	VEGF	TAM release TGF-β to downregulate miR-34a	Improve the proliferation and invasion of colorectal cancer (27)
MiR- 4319	Decreased	NECAB3	Promote M2 macrophage polarization	Promote Non-small cell lung cancer progression (45)
MiR-21	Increased	SNAI1 MRC1	Suppress M1 markers and enhance M2 markers	Promote tumor angiogenesis and growth (21)
MiR-101	Increased	DUSP1	Regulate macrophage to innate immune responses	Promote hepatocarcinoma growth and metastases (46)
MiR-222-3p	Increased	SOCS3	Induce polarization of the M2 phenotype	As a biomarker of epithelial ovarian cancer (47)
MiR-106b-5p	Increased	IRF1/IFN-β	Promote M2 polarization	Enhance glioma growth (48)
MiR-17 MiR-20a		HIF-2a	Enhance proangiogenic	Contribute to the angiogenic process within tumors (29)
MiR-30e*	Decreased	Bmi1	Enhance the regulation mediated by TAM	Promote tumor growth, invasion, and metastasis of gastrointestinal cancer (49)
MiR-146a MiR-222	Decreased	NF-κB p50 subunit	Promote the phenotype molecules of M2 macrophage Enhance TAM chemotaxis	Promote tumor growth (50)
MiR let-7b	Decreased		Modulate macrophage polarization	Reduce angiogenesis and prostate carcinoma (51)
MiR-125a/b	Decreased	CD90	Enhance the function of TAM	Promote HCC cell proliferation and stem cell properties (31)

### MiRNA Roles in DC

**Figure 2:**
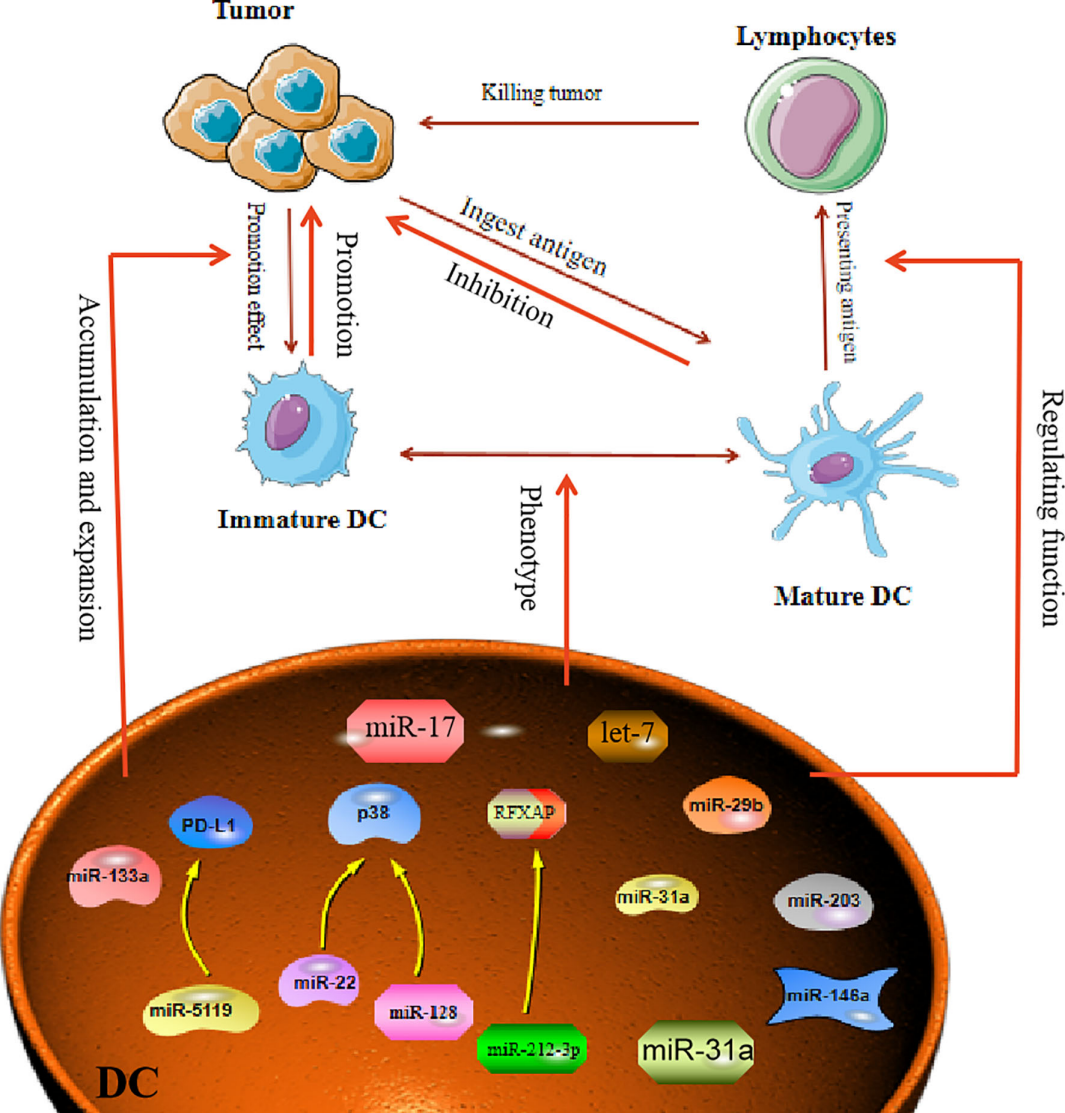
MiRNA affects tumor process by regulating Dendritic Cells. Mature dendritic cells can engulf and present tumor antigens to kill tumors. In the TME, dendritic cells often show immature phenotypes. MiRNA is involved in regulating the immature differentiation process of dendritic cells. At the same time, miRNA can also promote the transformation of immature phenotypic of DC cells, thereby affecting the proliferation and escape of tumor cells.

DC in the tumor environment have unique functions that distinguish them from normal DC, which usually promote tumor development. However, miRNA can affect the function and differentiation of DC. MiR-221, as an oncogene, can induce cell proliferation in HCC and inhibit the maturation of DC (52). In breast cancer, it was reported that miR-5119 was downregulated in splenic DC, and this led to decreased expression of PD-L1 and prevented T-cell exhaustion (53). Therefore, in the TME, when miRNAs are abnormally expressed in DC, the miRNAs will have a direct impact on the maturation and function of DC. Another study proclaimed that in an osteosarcoma mouse model, overexpression of miR-133a suppressed the maturation and activation of splenic DC (54). In addition, both miR-128 and miR-22 can affect the function of DC by targeting P38 (55). MiR-128 enhances the antitumor immunity of DC by targeting P38, while miR-22 damages the antitumor function of DC after targeting p38. Tumor-derived miRNA can also affect the function of DC. For example, transfection of DC with miR-203 inhibited the production of TNF-α and IL-12. TNF-α and IL-12 are known to strengthen the cross-presentation of DC. Therefore, inhibition of TNF-α and IL-12 production by miR-203 led to impaired antigen presentation in the DC (56). Moreover, miRNA influences the maturation of DC, as exemplified by miR-146a, which was demonstrated to regulate DC maturation and function. Treatment of DC with highly metastatic human pancreatic cancer cell line BxPC-3 conditioned medium (BxCM) resulted in upregulation of miR-146a and inhibition of Smad4 in the DC. Furthermore, BxCM stimulation of CD14+ monocyte-derived DC significantly reduced the differentiation and antigen presentation functions of DC (57). In addition to these effects, there are reports suggesting that tumor exosome-derived miRNA can affect DC functions. Pancreatic cancer (PC) -derived exosomal miR-212-3p can be transferred to DC to inhibit the expression of regulatory factor X-associated protein (RFXAP), which subsequently leads to a decline in MHC II expression in DC, thereby inducing immune tolerance in DC (58). In some cases, miRNAs in TME can reprogram DC functions. For example, multiple myeloma (MM) cells can induce DC with a tumor-permissive phenotype. The MM cells downregulate miR-29b in the DC, which leads to reprogramming and impairment of the DC functions and thus promotes growth and survival of MM cells (59). These facts all demonstrate that abnormally expressed miRNA in the tumor environment can affect the maturation and function of DC to promote or inhibit the tumor (**Figure 2**).

In the TME, miRNAs are also regarded as clinical prognostic molecules related to DC. In lung cancer, researchers found that lung tumor-infiltrating DC highly express PD-L1 molecules and have some molecular markers related to TAM. By analyzing the miRNAs in these tumor-infiltrating DC, miR-31a was found to play a significant role in promoting cancer, and overexpression of this miRNA could promote tumor invasion and metastasis. Therefore, the presence of miR-31a in tumor-infiltrating DC is seen as a poor prognostic indicator in NSCLC (60). MiR-17-5p derived from gastric cancer cells can serve as a biomarker for gastric cancer detection because when DC absorb miR-17-5p, this miRNA can inhibit the maturation of DC and weaken the antitumor effect of DC (61). In addition to these miRNAs that are already reported as clinical predictors, all miRNAs related to DC dysfunction can be regarded as having predictive functions for clinical treatment, and the identification of such miRNAs will facilitate the development of improved anticancer drugs.

In recent years, the role of DC-related vaccines in tumor treatment has been extensively studied, and certain drugs have entered phase I or phase II clinical trials. Designing a DC-based vaccine needs to ensure that the DC have a mature phenotype and can migrate to lymphoid organs to activate a more powerful protective immune response. As previously mentioned, miRNAs can affect the phenotype of DC. Assessment of hsa-miR-155, hsa-miR-146a, hsa-miR-125a-5p, and hsa-miR-29a in mature DC and immature DC revealed that these molecules had different distributions in different DC. Therefore, these miRNAs could be used to predict the feasibility of developing DC as a vaccine (62). Additionally, co-encapsulation of miR-148a inhibitor (miR-148ai) with poly I:C and OVA through cationic peptide micelles was used to generate an integrated peptide microgels/poly I:C(PMP)/OVA/148ai Nano vaccine. This vaccine could reprogram the function of tumor-infiltrating DC and increase DC numbers in the spleen and TME (63). These observations indicate that miRNA not only plays a guiding role in DC vaccines, but also can enhance the antitumor effect of DCs by combining both of them in some conditions.

The continuous understanding of miRNA has resulted in some miRNA-related drugs entering the clinical research phase. Studies have revealed that using small molecule mimics to treat tumors can alter the function of DC. For example, let-7 analogs synthesized by the nucleic acid delivery system can activate TLR-7 on the surface of DC and inhibit the production of IL-10 by DC. Then it can reactivate the function of DCs and reverse the tumor suppressive microenvironment to inhibit tumor growth (64).This demonstrates that with the help of miRNA regulatory effects on DC, miRNA can be designed as drugs to treat tumors.

### MiRNA Roles in NK Cells

**Figure 3:**
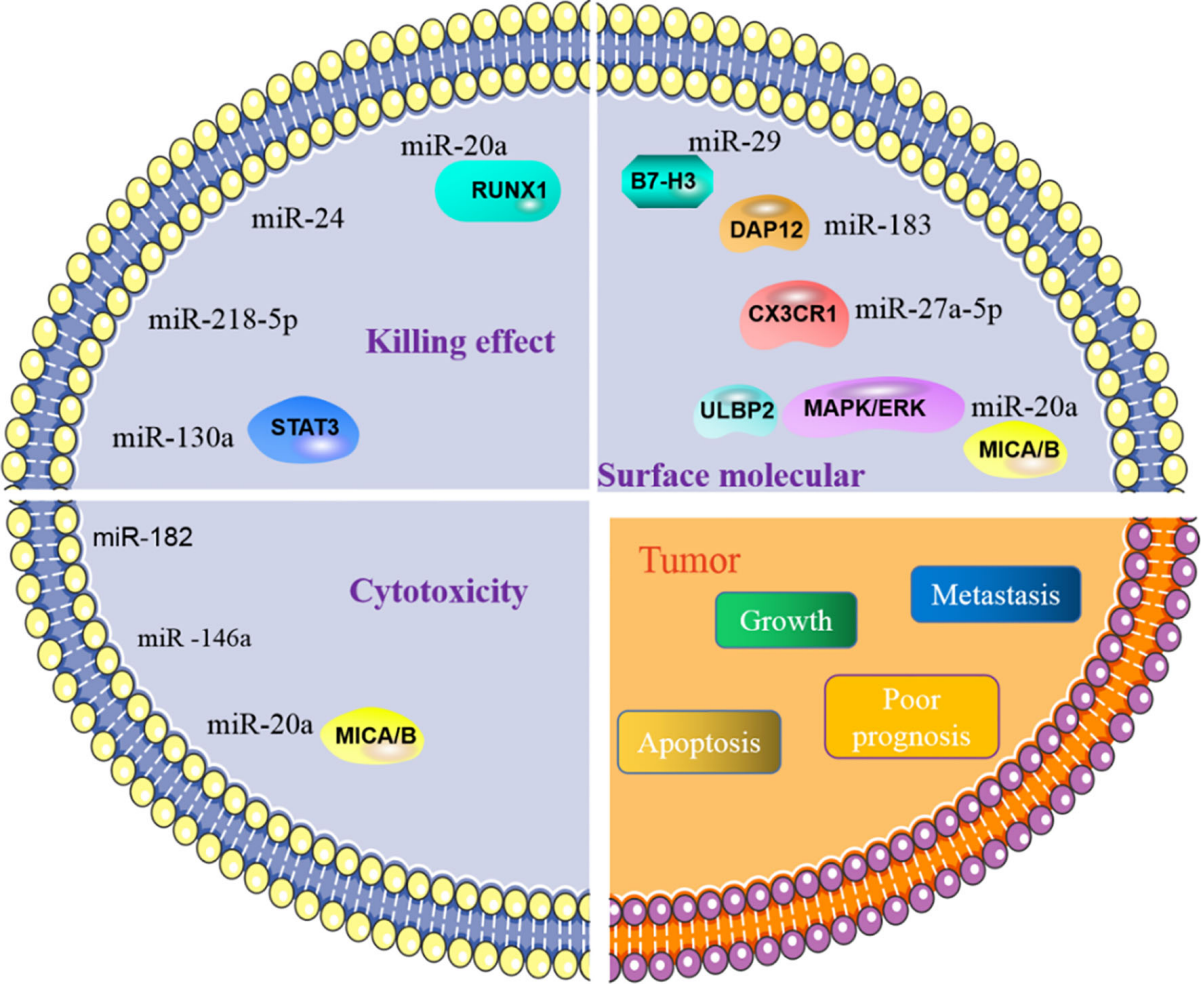
MiRNA affects tumor process by regulating NK cells. NK cells are primarily involved in killing tumors. The role of NK cells in killing tumors is manifested in killing effect and its cytotoxicity. MiRNA is involved in regulating the effect of NK cells on tumors, by directly or indirectly regulating the killing effect and cytotoxic function of NK cells. In addition, miRNA can also govern the activity of NK cells, affect the activation of NK cells and then control tumor behavior.

NK cells eliminate tumor antigens predominantly by producing numerous cytotoxic products such as perforin and granzyme. MiRNA is associated with regulating the killing and cytotoxic effects of NK cells (**Figure 3**), and this regulation is predominantly direct. Abnormal expression of miRNA in NK cells can affect the function of NK cells by targeting various downstream genes. In cervical cancer, miR-20a was upregulated in NK cells, and overexpression of miR-20a inhibited the killing effect of NK cells on tumor cells by targeting RUNX1 (65). Similar to miR-20a, miR-24 (66) and miR-218-5p (67) have also been proven to reduce the killing effect of NK cells in colorectal cancer and lung adenocarcinoma (LA), respectively. However, contrary to the functions of these miRNAs, some miRNAs can enhance the killing effect of NK cells. In NSCLC, miR-130a is significantly reduced in NK cells, but STAT3 is increased. Moreover, when miR-130a was overexpressed in NK cells, it enhanced the killing effect of NK cells by targeting STAT3 (68). MiRNA also directly regulates the cytotoxicity of NK cells. For example, miR-182 is highly expressed in NK cells of HCC, and co-culturing NK cells overexpressing miR-182 with Huh-7 (human hepatoma cell line) cells enhanced the cytolytic effect of the NK cells (69). Conversely, certain miRNAs can inhibit the cytotoxicity of NK cells. Overexpression of miR-146a in patients with chronic hepatitis B (CHB) and HCC can reduce NK cell-mediated cytotoxicity and the production of IFN-γ and TNF-α (70). In addition to these direct effects, studies have found that miRNAs abnormally expressed in tumor cells can indirectly affect the function of NK cells. For instance, overexpression of miR-20a in human ovarian cancer tissues is thought to enhance the proliferation and invasion of cancer cells. It was found that the highly expressed miR-20a in tumor cells can target the expression of MICA/B. MICA/B is usually produced by tumor cells and engages with the receptor NKG2D on the surface of NK cells, causing tumor cells to be recognized and killed by NK cells. Therefore, miR-20a targeting and silencing MICA/B can indirectly inhibit the cytotoxicity and tumor killing effects of NK cells (71). These findings indicate that in the TME, miRNA directly or indirectly affects the killing effect and cytotoxicity of NK cells by controlling the expression of various target genes. (**Table 2**).

**Table 2:** MiRNA related with NK cells in tumor progression.

MiRNA	Status	Target	MiRNA’s regulating in NK cells	Impact on tumor progression
MiR-20a	Elevated	RUNX1	Inhibit the killing effect	Promote cervical cancer cells growth (65)
MiRNA-130a	Elevated	STAT3	Potentiate the killing ability	Inhibit Non-small cell lung cells growth (68)
MiR-182	Elevated	NKG2D NKG2A	Enhance NK cell cytotoxicity	(69)
MiR-24	Elevated	Paxillin	Inhibit the killing effect	Increase tumor volume of colorectal cancer (66)
MiR-544	Elevated	RUNX3	Decrease IFN-γ secretion and the NK cell cytotoxicity	Promote liver cancer cells growth (72)
MiR-140-3p	Elevated	MAPK1	Inhibit the cytotoxicity	Facilitate ovarian cancer cells growth (73)
MiR-1245	Elevated		Downregulate NKG2D expression and impair NKG2D-mediated immune responses	NKG2D can promote NK cells activation to kill tumor (74)
MiR-20a	Elevated	MICA	Reduce MICA expression	Decrease Colorectal cancer cells sensitive to NK cells (75)
MiR-519a-3p	Elevated	ULBP2 MICA	Impair NK cells killing effect	Facilitate cancer progression and evasion (76)
MiR-146a	Elevated	STAT1	Reduce NK cell-mediated cytotoxicity	(70)
MiR-27a-5p	Elevated	CX3CR1	Decrease the accumulation of NK cells	Reduce the damage of NK cells to tumor cells (77)
MiR-30c-1*	Elevated	HMBOX1	Depress the activation of NK cells	Inhibit the development of liver cancer (78)
MiRNA-155	Elevated	BRG1	Control natural killer/T-cell lymphoma cells viability	Promote primary xenograft growth as well as tumor-associated lymphangiogenesis (79)
MiR-29	Decreased	B7-H3	Overexpression of B7-H3 reduces the cytotoxicity effect of NK cells	Promote immune escape of solid tumors (80)
MiR-17/20a	Elevated	Mekk2	Enhance NK cells to recognize tumor cells	Inhibit tumor development (81)
MiR-186	Elevated	MYCN	Enhance NK cell activity	Inhibit neuroblastoma growth and immune escape (82)
MiR-561-5p	Elevated	CX3CL1	Decrease CX3CR1+ NK cell infiltration and function	Promote tumorigenesis and metastasis (83)
MiR-506	Decreased	STAT3	Reduce NK cells cytotoxicity	Promote hepatocellular carcinoma cells progression (84)
MiR-218-5p	Elevated	SHMT1	Suppress the killing effect of NK cells	Reduce the killing effect on Lung adenocarcinoma (67)
miR-146b-5p	Elevated	WBSCR22	Enhance the function NK cells	Inhibit the oxaliplatin-resistant of colorectal cancer cell (85)
MiR-302c MiR-520c	Decreased	MICA/B ULBP2	Upregulate MICA/B and ULBP2 to active NK cells	Enhance the susceptibility of cancer cells to NK cells (86)

NK cells contain various molecular receptors that usually perform different functions. For example, the activation receptor NKG2D can initiate NK cell activity, while NKG2A as an inhibitory receptor can block the activation of NK cells. MiRNA can affect the activity of NK cells by controlling the expression and function of such receptors. NKG2D is one of the main activating receptors of NK cells and usually interacts with its ligands known as NKG2DLs. NKG2DLs, including MICA/B and ULBP1/2/3, are commonly expressed in tumor cells and their main role is to give effect to NK cells to eliminate tumors. In breast cancer, miR-20a was found to inhibit the expression of MICA/B and ULBP2 by directly targeting MICA/B and downregulating ULBP2 through inhibition of MAPK/ERK, which ultimately resulted in a decrease in the cytotoxicity of NK cells (87). In addition, NK cells express certain receptors related to the chemotactic recruitment of NK cells, which can drive the accumulation of these cells to tumor or local injury areas. CX3CR1 is one such chemotactic receptor on the surface of NK cells. In neuroblastoma, it was discovered that the proportion of CX3CR1 in NK cells was significantly reduced, while the amount of miR-27a-5p increased. Further investigation confirmed that miR-27a-5p was the principal regulator of CX3CR1. In addition to organizing the receptors on the surface of NK cells, miRNAs can indirectly influence the functions of NK cells by affecting some essential protein molecules in NK cells. MiR-183 induced by TGF-β can inhibit DNAX activating protein 12 (DAP12), a protein that is required to maintain the stability of NK cell surface receptors. Therefore, when DAP12 is depleted, the function of NK cells is impaired (88). Furthermore, immunomodulatory molecule B7-H3, which inhibits NK cell activity, was confirmed to be governed by miR-29. However, in some TMEs, miR-29 is inhibited and consequently B7-H3 expression is significantly enhanced and can inhibit the activity of NK cells and limit tumor damage (80).

The regulation of NK cells by miRNA is closely related to some behaviors of tumor cells, including tumor proliferation, metastasis, and immune escape. For instance, miR-186 transported by NK cell exosomes could reduce the growth ability of MYCN-amplified neuroblastoma cells and prevent immune suppression mediated by TGF-β1 (82). Tumor metastasis is impacted by the effect of some miRNAs on NK cells. For example, miR-296-3p is highly expressed in human prostate cancer and can directly inhibit the expression of intercellular adhesion molecule 1 (ICAM-1). High expression of miR-296-3p can increase the resistance of circulating tumors to NK cells, thereby improving the metastasis ability of tumor cells (89). The apoptosis process of tumor cells is also related to miRNA and NK cells. High expression of miR-519a-3p in breast cancer was shown to not only induce the expression of TRAIL and FasL to increase the apoptosis resistance of tumor cells, but also reduced the sensitivity of tumor cells to NK cells by downregulating the expression of the NKG2D ligands ULBP2 and MICA. Furthermore, this combination of activities ultimately encourages tumor cell growth (76). In addition, miRNA is an indicator of poor prognosis associated with NK cells in TME. For instance, miR-183 is significantly increased in renal cell carcinoma (RCC), and it was confirmed that when serum miR-183 is at a low level, the sensitivity of RCC to NK cells is enhanced. Therefore, miR-183 can be used as a detection index to predict the toxic function of NK cells (90). In summary, miRNA regulation of NK cells is also associated with the process of regulating tumor cells.

### MiRNA Roles in MDSC

Numerous studies have demonstrated that miRNA is involved in the differentiation, maturation, and function of MDSC. This is mainly reflected by the observations that abnormal expression of miRNA in tumor cells or MDSC induced by the tumor environment affect the processes of MDSC generation, differentiation, amplification, and function, and thereby influence tumor progression.

**Figure 4:**
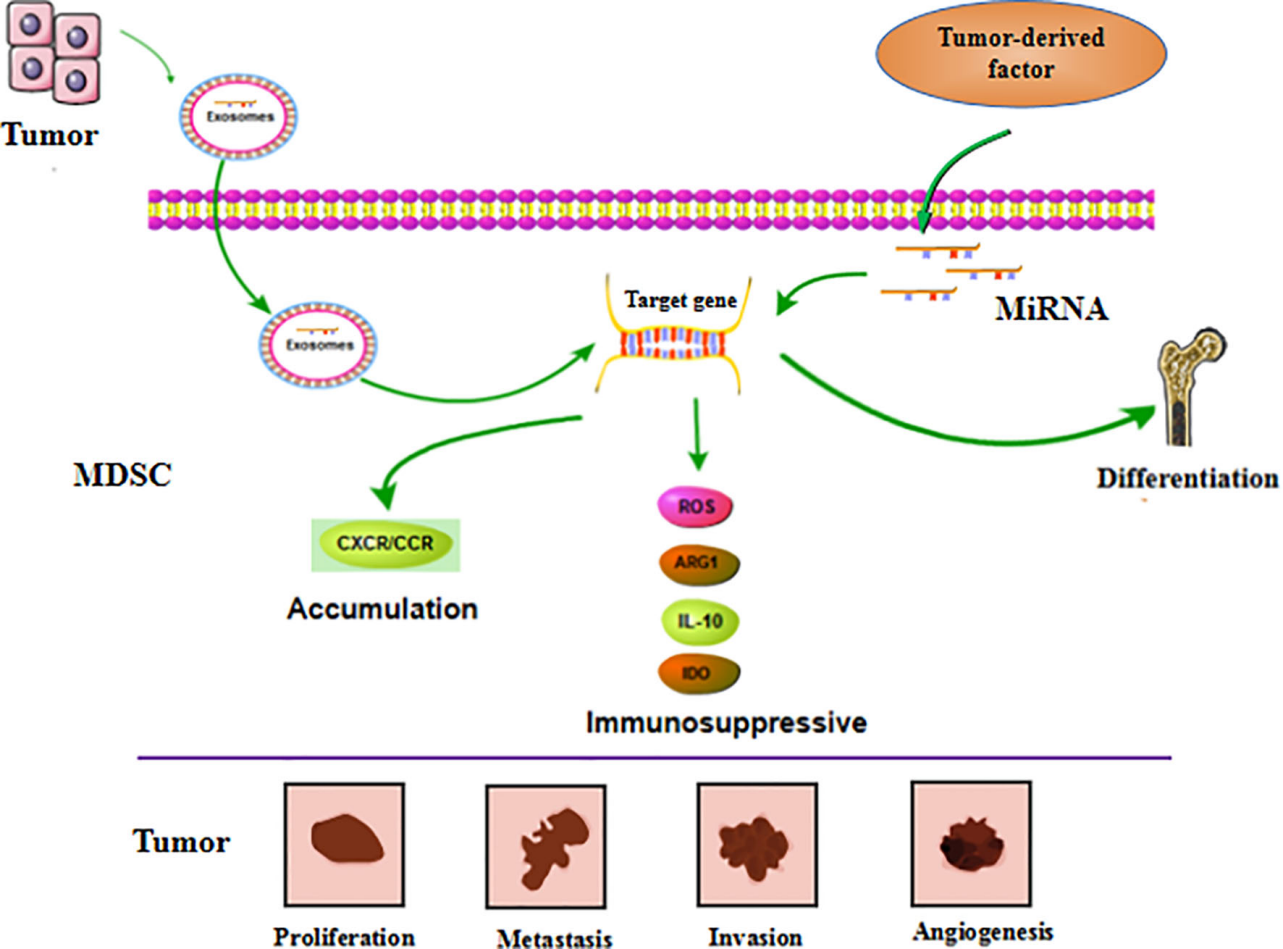
MiRNA affects tumor progression by regulating MDSC. MiRNA is involved in regulating the differentiation, accumulation and function of MDSC. These miRNAs can be derived from MDSC itself. Furthermore, it’s also generated by tumor cells and transported to MDSC through exosomes to exert function. These regulatory effects ultimately affect tumor cell proliferation, metastasis, invasion, and vascular proliferation.

MiRNAs in tumor cells mainly use exosomes to influence the tumor process and regulate the production, aggregation, and function of MDSCs (**Figure 4**). In breast cancer, tumor exosome-derived miR-9 and miR-181a can metastasize to MDSC to promote the early development of these cells by interfering with SOCS3 and PIAS3. Moreover, when these two miRNAs are transferred to MDSC, they can inhibit the proliferation of T cells, activate apoptosis of T cells, and accelerate tumor growth (91). Economic miRNA produced by gastric cancer cells was also reported to impact MDSCs. MiR-107 was shown to not only be overexpressed in gastric cancer cells but was also enriched in tumor-derived exosomes. These miR-107-rich tumor-derived exosomes absorbed by HLA-DR CD33+ MDSC could target and inhibit the expression of DICER1 and PTEN genes, and subsequently promote the amplification of MDSC (92). These findings confirm that miRNAs in tumor cells play a regulatory role in MDSC. The regulatory effects are predominantly indirect, affecting the infiltration and function of MDSC in tumors by means of cytokines or exosomes.

MiRNA in MDSC can also affect the function and differentiation of MDSC (**Figure 4**). The TME is known to lead to massive generation and aggregation of MDSCs. Studies have shown that after miRNAs were abnormally expressed in MDSCs, they would play different roles depending on their expression. Overexpression of miRNA such as miR-30a in MDSC can promote infiltration of MDSC into tumors. In mouse B-cell melanoma, granulocytic MDSC (G-MDSC) and monocytic MDSC (M-MDSC) highly express miR-30a (93), which can further upregulate ARG1, IL-10, and ROS to promote the differentiation of MDSC and increase tumor growth. Other researchers found that in lung cancer, miR-486 was highly expressed in tumor-induced MDSCs and could inhibit the apoptosis of bone marrow cells and promote tumor metastasis (94). MiR-9 is also reported to impact MDSC and promote tumor formation. Inhibition of miR-9 promoted differentiation of MDSC and significantly reduced the immunosuppressive function (95). Congruent with the high expression of these miRNAs promoting the differentiation and function of MDSC, downregulation of some miRNAs has been shown to accelerate the infiltration of MDSC in tumors. For example, the absence of miR-155 in MDSC can promote MDSC infiltration in tumors and can advance the formation of tumor blood vessels and promote the generation of MDSC (96). Tumor-promoting PMN-MDSC and M-MDSC both have low expression of miR-223, which is supervised by tumor-related factors. In the presence of tumor-related factors, miR-223 can target MEF2C to inhibit tumor-induced MDSC differentiation from bone marrow cells, and then suppress tumor growth (97). Some miRNAs appear to exhibit dual regulatory effects, usually displaying oncogenic or antitumor effects. However, there are currently no detailed reports on these observations. One example of a miRNA with dual regulatory effects is miR-155, which has altered expression in diverse tumors. High expression of miR-155 can promote MDSC infiltration in tumors, but minimal expression of miRNA-155 conversely generates immunosuppressive functions in MDSC. Knowledge on these dual regulatory effects will be updated with the continuous advancement of science.

Cytokines or inflammatory factors produced by tumor cells are concluded to affect MDSC. Tumor-derived TGF-β1 upregulated miR-494 in MDSC to target PTEN (phosphatase and tension homolog) and activate the PI3K/Akt pathway. This enhanced the immunosuppressive activity of MDSC, and the enhanced expression of metalloproteinases (MMPs) promoted the escape and metastasis of tumor cells (98). Moreover, hypoxia induced high expression of miR-210 in MDSC, and thereby enhanced MDSC promotion of tumor progression (99). However, the effect of miRNA in MDSC can also be mediated by exosomes. When miRNAs are generated in MDSC, they can be transmitted to other cells through exosomes and subsequently affect the TME. Breast tumor cells treated with DOX were observed to release IL-33, which induced IL-13+ Th2 cells to produce IL-13. The produced IL-13 promoted the generation of DOX-MDSC and MDSC miR-126a+ exosomes through MDSC IL-13R (100), and this promoted the proliferation of tumor blood vessels and strong inhibition of Th1 cells, and finally enhanced lung metastasis.

In summary, these research findings show that tumor cells or MDSC abnormally expressing miRNA can accelerate the formation of MDSC in the TME and enhance the immunosuppressive activity of MDSC. Thus, miRNA has a critical role in the function and development of MDSC in the TME (**Table 3**).

**Table 3:** MiRNA related with MDSC in tumor progression.

MiRNA	Status	Target	MiRNA’s regulating in MDSC	Impact on tumor progression
MiR-30a	Upregulated	SOCS3	Increase differentiation and immunosuppressive function	Facilitate tumor growth of B-cell lymphoma (93)
MiR-494	Upregulated	PTEN	Enhance accumulation and functions	Promote tumor invasion and metastasis by upregulating MMPs (98)
MiR-155 MiR-21	Upregulated	SHIP-1 PTEN	Boost expansion	Accelerate tumor growth (101)
MiR-155	Downregulated	HIF-1α	Enhance the recruitment and functions	Promote tumor growth and angiogenesis (96)
MiR-486	Upregulated	Cebpa	Promote proliferation and inhibit apoptosis	(94)
MiR-223	Downregulated	MEF2C	Suppress differentiation	Reduce tumor growth (97)
MiR-200c	Upregulated	PTEN FOG2	Promote suppressive potential	Promote tumor growth (102)
MiR-17-5p MiR-20a	Downregulated	STAT3	Alleviate the suppressive function	Reduce tumor growth (103)
MiR-9	Upregulated	Runx1	Inhibit the differentiation and enhance function	Promote tumor growth (95)
MiR-9 MiR-181a	Upregulated	SOCS3 PIAS3	Promote early-stage development	Promote tumor growth and immune escape (91)
MiR-126a	Upregulated	S100A8/A9	Promote expansion	Promote tumor angiogenesis and lung metastasis (100)
MiR-29a MiR-92a	Upregulated	Hbp1 Prkar1a	Enhance the proliferation and function	(104)
MiR-107	Upregulated	DICER1 PTEN	Induce the expansion and activation	Facilitate invasion and metastasis (92)

### MiRNA Roles in Treg

In the previous chapters, we have summarized the specific functions and characteristics of Treg in the TME. Studies have found that miRNA are engaged in the process of regulating Treg in the TME, reflected mainly through miRNA affecting the differentiation, function, and aggregation of Treg in the TME. In addition, other studies have shown that miRNA also play an important role in the regulation and balance of Treg and their effect on tumor growth.

**Figure 5:**
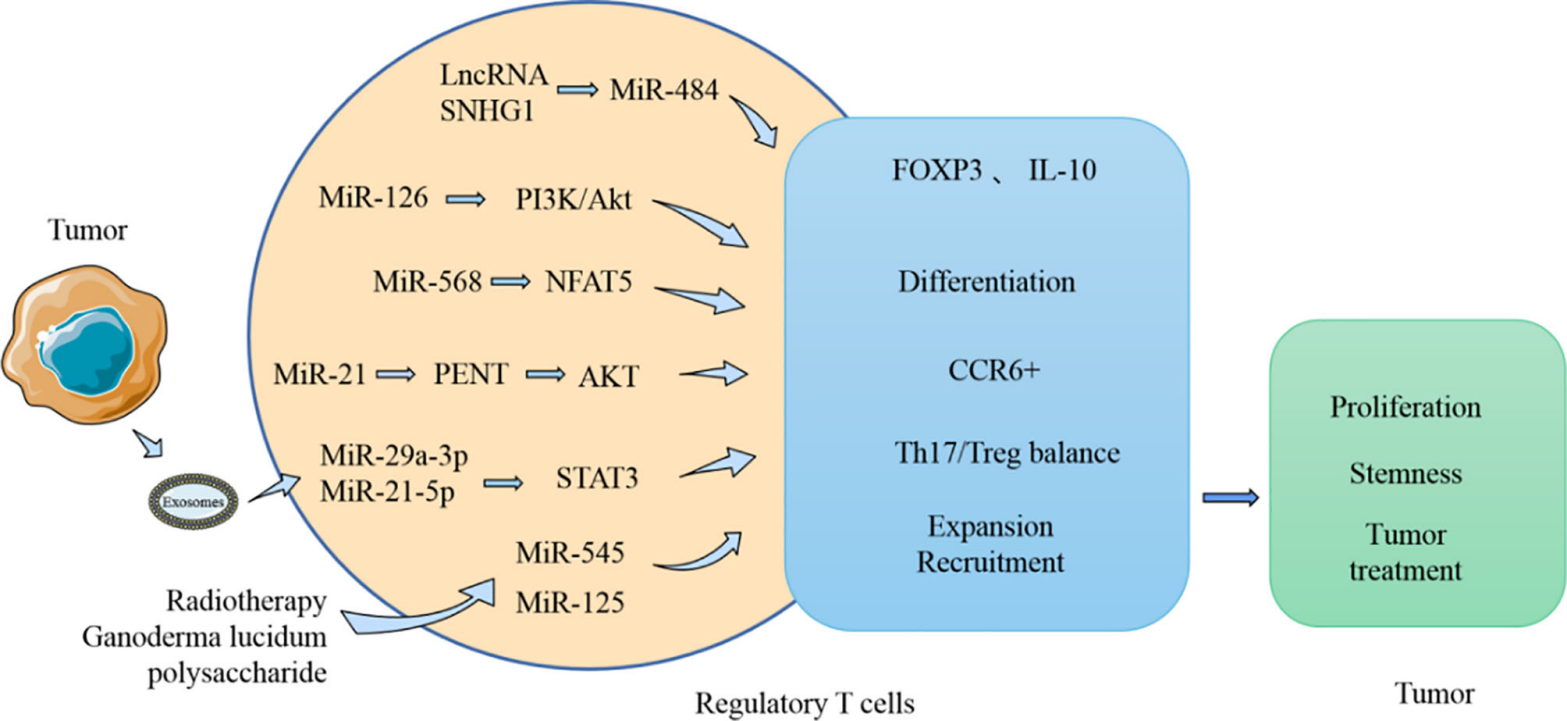
MiRNA influences the tumor process by modulating Treg. MiRNA participates in the regulation of the production and function of Treg in TME. MiRNA can affect the expression of FOXP3 and IL-10, which in turn affects the activity of Treg on tumor. Moreover, the Th17/Treg balance regulated by miRNA can also influence tumor development.

MiRNA can affect the differentiation and function of Treg (**Figure 5**). Treg are usually differentiated from resting CD4+ T cells, and FOXP3 is considered a characteristic biomarker of Treg. Recent studies have found that LncRNA SNHG1 is highly expressed in CD4+ tumor-infiltrating lymphocytes of breast cancer and can inhibit the expression of miR-484. In addition, FOXP3 and IL-10 expression can be reduced by using siRNA-SNHG1. Therefore, the authors concluded that inhibiting SNHG1 inhibits Treg differentiation, and this effect is probably achieved by SNHG1 through regulation of miR-484 (105). MiR-586 and miR-126 are thought to have regulatory effects on induction of Treg differentiation. MiR-568 can inhibit Treg activation and function by targeting NFAT5 (106). However, miR-126 overexpression induces the generation of Treg and enhances the function of these cells by targeting p85b and affecting the PI3K/Akt pathway (107). These two miRNAs both affect the function of Treg, with miR-586 inhibiting the immunosuppressive function of Treg. However, it has been noted in breast cancer models that when miR-126 is silently expressed, the immunosuppressive function of Treg is impaired, and the antitumor activity of CD8+ cells is enhanced. MiRNAs are also involved in other functions of Treg in the TME. Tumor-derived miR-214 is reported to be transported to CD4+ T cells and promotes the expansion of Treg by downregulating the expression of PENT. These miR-214-induced Treg promote tumor growth and proliferation by producing IL-10 (108). In addition, elevated expression of miR-124 in glioma stem cells inhibited the expression of STAT3 and reversed the inhibitory effect of glioma stem cells on the proliferation of T cells and the induction of Treg (109). The expansion and accumulation of Treg can be impacted by miRNA. CCR6+ Foxp3+ Treg are usually found in large numbers in the TME and are regarded as related to the poor prognosis of patients with breast cancer. MiR-21 is highly expressed in CCR6+ Tregs in tumor tissues of patients with breast cancer. Moreover, *in vitro* experiments demonstrated that miR-21 could inhibit the proliferation of CCR6+ Tregs, mainly through targeting PENT and then activating the AKT pathway (110).

MiRNA also affect the balance of Th17/Treg (**Figure 5**). Resting CD4+ T cells are known to generate Th1, Th2, Th17, and Treg cells after differentiation, and these cells usually perform distinct immunological functions. The Th1 type immune response is beneficial for accelerating the elimination of foreign pathogens. However, the Th2 type immune response usually produces numerous inflammatory factors and accelerates the development of certain diseases. Simultaneously, Th17 cells, which are instrumental in the inflammatory response, are in equilibrium with Treg cells in a stable environment. The process of Th17/Treg imbalance mediated by PD-1/PD-L1 was found to be affected by miR-21 (111). After patients with gastric cancer undergo resection, the number of Th17 cells decreases, and the number of Treg cells and PD-1/PD-L1 expression levels also both reduce. Silencing PD-1 expression *in vitro* using Ad-sh-PD1 promoted the expression of miR-21 and enhanced the percentage of Th17 cells, but decreased the percentage of Treg cells. However, overexpression of miR-21 can increase the percentage of Th17 cells and decrease the percentage of Treg cells. Therefore, miR-21 affects the balance between the Th17/Treg, leading to an imbalance in their interaction. Furthermore, exosomes produced by TAM can carry miR-29a-3p and miR-21-5p, and when these two miRNAs are absorbed by CD4+ T cells, they can inhibit STAT3 and induce an imbalance between Th17/Treg (112); a high proportion of Treg/Th17 usually promotes the growth and metastasis of ovarian cancer.

In addition to the above effects, miRNAs are involved in some treatments that interfere with Treg function and activity. In the treatment of Lewis lung tumor with radiotherapy, tumor-infiltrating CD4+CD25+ Treg and CCL2 levels decrease, while miR-545 levels increase. Concurrently, silencing miR-545 was found to promote the proliferation of CD4+CD25+ Treg and reduce the sensitivity of cancer cells to radiotherapy. Therefore, radiotherapy can inhibit the expansion and recruitment of Treg by upregulating miR-545 (113). Ganoderma lucidum polysaccharide is a traditional medicine with immunomodulatory and antitumor effects. Ganoderma lucidum polysaccharides were revealed to inhibit the proliferation of liver cancer cells. Simultaneously, T cells treated with Ganoderma lucidum polysaccharide were discovered to enhance the expression of miR-125 and thereby inhibit the expression of FOXP3. Therefore, Ganoderma lucidum polysaccharide could block the inhibitory effect of Treg on effector T cells (114). In conclusion, Treg as a class of immunosuppressive cells, are regulated by miRNA, and every process of tumor development can be affected by interfering with the functions and other behavioral characteristics of Treg.

### MiRNA Roles in Granulocytes

MiRNAs are proposed to be involved in the regulation of granulocytes, but reports on this are rare. It has been acknowledged that in the inflammatory environment, miRNA can affect the activation, aggregation, and function of granulocytes. However, there are limited reports on the regulation of granulocytes by miRNA in cancer. MiR-223 has been established to affect the differentiation process of neutrophils, and other studies have suggested that miR-223 can affect the accumulation of neutrophils in the lung through CXCL2 and CCL3 (115). MiR-142, miR-466l, and miR-451 were shown to participate in the process of granulocytes. MiR-142 promoted the maturation and differentiation of myeloid cells into granulocytes and monocytes (116), while miR-451 reduced the recruitment and infiltration of neutrophils by downregulating the p38 MAPK signaling pathway (117). It can thus be concluded that miRNA can affect the initiation, differentiation, recruitment, and function of granulocytes, especially neutrophils. However, there are very few reports at present that link miRNAs to the characteristics of neutrophils in cancer.

Some reports have suggested that miRNA can influence the behavior of tumor cells by regulating granulocytes. Among them, tumor-related neutrophils are considered to play a major role in the tumor environment. The distribution of miRNAs in N2 neutrophils of colon cancer were analyzed, and through software prediction and experiments, hsa-miR-4780 was found to be upregulated and hsa-miR-3938 was downregulated in N2 neutrophils compared with neutrophils. Moreover, hsa-miR-4780 and hsa-miR-3938 could target and regulate TUSC1 and ZNF197, respectively. Therefore, it is believed that hsa-miR-3938 and hsa-miR-4780 may have a role in regulating the invasion and metastasis of colon cancer by controlling N2-type neutrophils (118). Certain products generated by neutrophils can also promote tumors. Neutrophil gelatinase-associated lipocalin (NGAL) is a small molecule protein that is usually related to the epithelial mesenchyme of tumor cells. MiR-138 can regulate the expression of NGAL in various tumor cell lines, such as breast, endometrial, and pancreatic carcinomas. When pancreatic cancer cells were transfected with miR-138, NGAL was inhibited and the metastasis and proliferation of tumor cells were also suppressed (119). Therefore, miRNAs can govern the proliferation, metastasis, and escape of tumor cells by manipulating neutrophils. Further research on the function of neutrophils is likely to uncover some additional behaviors of tumor cells influenced by neutrophils.

## MiRNA-Based Drugs

The development of miRNA as a clinical drug is undoubtedly imminent. Currently, it is believed that there are two main strategies for developing miRNA as therapeutics (120). Firstly, for miRNAs with cancer-inhibiting effects, miRNA analogs can be delivered through the system to promote their long-lasting and efficient anticancer effects (121). The second strategy is for oncogene miRNAs and involves the use of antisense oligonucleotides (ASO), locked nucleic acids (LNA), or antagomiRs to block the effects of miRNA (121). Obviously, miRNAs with pharmaceutical value must have strong stability and low toxicity, and identification of a suitable delivery system is a crucial task. A commonly used method is to load miRNAs into nanoparticles (122). However, some other delivery systems were demonstrated to have the capability to deliver miRNAs. The neutral liposome 1, 2-dioleoyl-sn-glycero-3 phosphatidylcholine could deliver miRNA analogs in preclinical trials (123). Simultaneously, according to the characteristics of miRNA, the system of transmitting siRNA has been proposed to be used to transmit miRNA, including synthetic polyethylenimine, dendrimers, cyclodextrin, etc (124).

At the time of writing, numerous kinds of miRNAs are entering the clinical research stage. MRX34 is a lipid carrier that encapsulates miR-34 analogues in NOV40. MRX34 entered a multicenter phase I trial in 2013 for patients with primary liver cancer, small cell lung cancer, lymphoma, and multiple myeloma or renal cell carcinoma (125). Concurrently, the use of miR-16 analogue therapeutics entered phase I trials in patients with malignant pleural mesothelioma or NSCLC (126). Subsequently, a LNA-based antimiR-155 (MRG-106; miRagen Therapeutics) study for skin T-cell lymphoma and mycopathic fungal subtype has entered phase I clinical trials (127). The continuous exploration of miRNAs will lead to increasing numbers of miRNA-based drugs reaching the preclinical development stage.

MiRNAs have complex and extensive functions in cancer, which arouses interest in developing miRNAs as clinical therapeutics. There are some advantages to using miRNA as a drug. Firstly, miRNA has a wide range of functions. Studies have indicated that a single miRNA may have multiple biological functions and can simultaneously inhibit multiple biological behaviors of tumor cells. When developed as a drug, such miRNAs may have multiple effects. Secondly, miRNA drugs are small-molecule biopharmaceuticals, and therefore have the advantages of biopharmaceuticals, that is, the characteristics of a specific target and low toxicity. In addition, miRNA itself acts as a mediator. It can not only directly target and regulate the expression of downstream genes, but can also be regulated by certain upstream RNAs. This undoubtedly expands our search for tumor treatment strategies by using multiple methods. Such adjuvant therapy is expected to vastly improve the efficacy of tumor treatment. Unfortunately, although the development of miRNA drugs is progressing, there are still significant limitations in the development of miRNAs as drugs. These limitations are not only caused by miRNA itself, but also by the complexity of the drug development process. Nucleotides of small molecules have poor stability; therefore, the primary consideration in developing miRNAs as drugs is to how to maintain the stability of the miRNAs. This has led to more and more researchers turning their perspectives to the research of miRNA delivery vectors. In addition, selecting the appropriate route of administration, the drug reagent form, and the action process are also challenges in miRNA drug development. Furthermore, a significant drawback in using miRNA as a drug is that it can produce off-target effects. Therefore, the stable release of miRNAs to exert efficacy will also restrict the development of miRNA drugs.

## Conclusion

MiRNA exhibited impact on a variety of disease processes, especially inflammation and cancer in recent studies. It has been observed that immune cells in the TME not only fail to play a valid anti-tumor effect, but also promote oncogenesis. And miRNA has been considered as a significant molecular mechanism for the crosstalk between tumor cells and immune cell in the TME. Here we mainly discuss the impact of the miRNA on the TME and biological behavior of tumors, which can provide clearer information to further explore the relationship between miRNA and TME in the future. Meanwhile, we epitomized the effects of immune cells whose functions and properties are widely regulated by miRNAs on tumor behavior more comprehensively. These cells primarily embody macrophages, MDSC, DC and NK cells. Furthermore, we also declared a corresponding discussion of poorly studied miRNA-associated immune cells in detail, for example Treg cells and granulocyte. These suggest that the influence of miRNAs on the TME may cover an extensive range. And recent reports indicated that subsequent studies would turn attention to granulocytes and Treg cells to deeply explore the role of miRNAs in them to affect tumors. Similarly, there are few reports of miRNA on T lymphocytes and B lymphocytes to influence tumors. Our summary may light up some thoughts to investigate the mechanism of miRNA in lymphocytes and tumors. To sum up, it is ponderable for us to further comprehend the biology of tumors by penetratingly investigating the impact of miRNA on the TME. In addition, our review may have consulted implication for the tumor immunotherapy.

MiRNA’s regulation on the TME and tumor biological processes determines its momentous position in tumor therapy, especially in tumor immunotherapy. Tumor immunotherapy, including immune checkpoint inhibitor CART cell therapy, and cytokine therapy, has already been applied in the clinic, and DC vaccines are also utilized to treat tumors. It is regretful that rare miRNA drugs which show influence on tumor immunotherapy have entered into the preclinical research. However, a growing number of nucleic acid drugs have stepped in the stage of clinical research, taking siRNA drugs and mRNA drugs for instance. Studies have found that siRNA drugs are adopted to treat certain neurological diseases, while mRNA drugs are mainly put to express virus or tumor antigens. It is worth noting that more and more studies have proposed that mRNA drugs have peculiar positions in tumor immunotherapy. They can be utilized as tumor vaccines as well as immunomodulators. In addition, some researchers proclaimed that mRNA antibodies have a short half-life and are easy to be eliminated *in vivo*. In connection with these aspects, it also confirms that the exploitation of miRNA drugs have a promising future. Besides, based on the role of miRNA in the TME, it is valuable to develop miRNA as a drug for synergistic tumor immunotherapy. Such application could improve the efficacy of single treatments and suppress the rate of tumor survival. Concurrently, immune regulation by miRNA may overcome the phenomenon of foreign body rejection in immunotherapy. In brief, the era of small molecule RNA drugs is coming, and we believed that with the continuous exploration, miRNA drugs will be increasingly employed in the clinical therapeutics.

## Author Contributions 

GR and HN wrote this paper.They made equal contributions to the article. HQ, SC, XG, JS, YZ, XT and LZ completed the work of article data query. YX reviewed this paper. All authors contributed to the article and approved the submitted version.

## Funding

This work was supported by National Natural Science Foundation of China (No. 81971562), National Key Research and Development Project (No. 2017YFD0400303).

## Conflict of Interest

The authors declare that the research was conducted in the absence of any commercial or financial relationships that could be construed as a potential conflict of interest.
